# Collective Investigation of the Influence of the Silver Nitrate Injections on Phthisis

**Published:** 1901-09

**Authors:** Thomas J. Mays

**Affiliations:** Philadelphia, Pa.


### CORRESPONDENCE.

### Collective Investigation of the Influence of the Silver Nitrate Injections on Phthisis.

Philadelphia, Pa., August 15, 1901.

*To the Editor Buffalo Medical Journal :*

In 1892 the undersigned began a collective investigation of the action of cold in the treatment of acute pneumonia, and there is reason for believing that this procedure which resulted in gathering 400 cases of this disease thus treated, with a death-rate not quite 5 per cent., was an important factor in calling attention to the utility of that treatment, and in introducing it to the profession of this country. That research was based on the conviction that no remedy can be called truly successful until it has passed the exacting crucible of clinical experience, and it is now proposed to apply the same ordeal to the silver injection treatment of phthisis, which, in a large hospital, dispensary and private practice, reaching over a period of three years, and during which many thousand injections were administered, has given me greater satisfaction than any other method that I have ever employed. In keeping with the above expressed feeling, a cordial invitation is herewith extended to those members of the profession who have the inclination and opportunity to investigate this method of treating phthisis and to whom a reprint on the subject with full information and blanks to report cases, will be cheerfully sent on application.

Thomas J. Mays, M. D.

1829 Spruce Street.

